# Reduced Insulin Sensitivity Is Related to Less Endogenous Dopamine at D_2/3_ Receptors in the Ventral Striatum of Healthy Nonobese Humans

**DOI:** 10.1093/ijnp/pyv014

**Published:** 2015-03-24

**Authors:** Fernando Caravaggio, Carol Borlido, Margaret Hahn, Zhe Feng, Gagan Fervaha, Philip Gerretsen, Shinichiro Nakajima, Eric Plitman, Jun Ku Chung, Yusuke Iwata, Alan Wilson, Gary Remington, Ariel Graff-Guerrero

**Affiliations:** Research Imaging Centre, Centre for Addiction and Mental Health, Toronto, Ontario, Canada (Mr Caravaggio, Ms Borlido, Ms Feng, Dr Gerretsen, Dr Nakajima, Mr Plitman, Mr Chung, Dr Iwata, Dr Wilson, Dr Remington, and Dr Graff-Guerrero); Institute of Medical Science (Mr Caravaggio, Dr Hahn, Mr Fervaha, Dr Gerretsen, Mr Plitman, Mr Chung, Dr Wilson, Dr Remington, and Dr Graff-Guerrero), and Department of Psychiatry (Drs Hahn, Gerretsen, Nakajima, Iwata, Wilson, Remington, and Graff-Guerrero), University of Toronto, Toronto, Ontario, Canada.

**Keywords:** dopamine, insulin, glucose, diabetes, D2

## Abstract

**Background:**

Food addiction is a debated topic in neuroscience. Evidence suggests diabetes is related to reduced basal dopamine levels in the nucleus accumbens, similar to persons with drug addiction. It is unknown whether insulin sensitivity is related to endogenous dopamine levels in the ventral striatum of humans. We examined this using the agonist dopamine D_2/3_ receptor radiotracer [^11^C]-(+)-PHNO and an acute dopamine depletion challenge. In a separate sample of healthy persons, we examined whether dopamine depletion could alter insulin sensitivity.

**Methods:**

Insulin sensitivity was estimated for each subject from fasting plasma glucose and insulin using the Homeostasis Model Assessment II. Eleven healthy nonobese and nondiabetic persons (3 female) provided a baseline [^11^C]-(+)-PHNO scan, 9 of which provided a scan under dopamine depletion, allowing estimates of endogenous dopamine at dopamine D_2/3_ receptor. Dopamine depletion was achieved via alpha-methyl-para-tyrosine (64mg/kg, P.O.). In 25 healthy persons (9 female), fasting plasma and glucose was acquired before and after dopamine depletion.

**Results:**

Endogenous dopamine at ventral striatum dopamine D_2/3_ receptor was positively correlated with insulin sensitivity (*r*(7)=.84, *P*=.005) and negatively correlated with insulin levels (*r*(7)=-.85, *P*=.004). Glucose levels were not correlated with endogenous dopamine at ventral striatum dopamine D_2/3_ receptor (*r*(7)=-.49, *P*=.18). Consistently, acute dopamine depletion in healthy persons significantly decreased insulin sensitivity (*t*(24)=2.82, *P*=.01), increased insulin levels (*t*(24)=-2.62, *P*=.01), and did not change glucose levels (*t*(24)=-0.93, *P*=.36).

**Conclusion:**

In healthy individuals, diminished insulin sensitivity is related to less endogenous dopamine at dopamine D_2/3_ receptor in the ventral striatum. Moreover, acute dopamine depletion reduces insulin sensitivity. These findings may have important implications for neuropsychiatric populations with metabolic abnormalities.

## Introduction

The continuous increase in the prevalence of obesity and diabetes in North America, thought to be linked to the overconsumption of high-fat/high-sugar foods, poses a serious public health burden (Mokdad et al., 2001; Seaquist, 2014). The concept of food addiction, where highly palatable foods are seen as rewarding as drugs of abuse (Lenoir et al., 2007), remains a hotly debated topic (Ziauddeen et al., 2012; Volkow et al., 2013a). In vivo brain imaging studies in humans have supported this concept, demonstrating similar brain changes between obese persons and persons with drug addiction (Volkow et al., 2013a, 2013b). More specifically, it has been demonstrated using positron emission tomography (PET) that obese persons and persons with drug addiction have less dopamine D_2/3_ receptor (D_2/3_R) availability in the striatum (Wang et al., 2001), an addiction-like neural marker also observed in rodents that overconsume palatable foods (Johnson and Kenny, 2010).

Striatal dopamine, particularly in the ventral striatum (VS), is an important modulator of food and drug reward and consumption (Palmiter, 2007). Several lines of evidence suggest that diabetes and reduced insulin sensitivity (IS) may be related to diminished endogenous dopamine in the VS. Reduced brain dopaminergic activity has been observed in diabetic rodents and postmortem human brains, as indicated by reduced dopamine synthesis rates (Crandall and Fernstrom, 1983; Trulson and Himmel, 1983; Saller, 1984; Bitar et al., 1986; Bradberry et al., 1989; Kono and Takada, 1994) and metabolism (Saller, 1984; Kwok et al., 1985; Bitar et al., 1986; Kwok and Juorio, 1986; Lackovic et al., 1990; Chen and Yang, 1991; Lim et al., 1994). Rodents made hypoinsulinemic via streptozotocin demonstrate reduced basal levels of dopamine in the nucleus accumbens (Murzi et al., 1996; O’Dell et al., 2014) as well as blunted dopamine release in response to amphetamine (Murzi et al., 1996; O’Dell et al., 2014). Notably, insulin modulates the cell surface expression (Garcia et al., 2005; Daws et al., 2011) and function (Owens et al., 2005; Sevak et al., 2007; Williams et al., 2007; Schoffelmeer et al., 2011) of the dopamine transporter (DAT). Moreover, insulin receptors are expressed in the nucleus accumbens and in midbrain dopaminergic neurons (Werther et al., 1987; Figlewicz et al., 2003), where they can modulate neuronal firing, energy homeostasis, and behavioral responses to rewarding stimuli like food, cocaine, and amphetamine (Galici et al., 2003; Konner et al., 2011; Schoffelmeer et al., 2011; Mebel et al., 2012; Labouebe et al., 2013). Collectively, these data suggest that decreased IS may be related to lower levels of endogenous dopamine in the VS.

To date, 2 PET studies have investigated the relationship between striatal dopamine D_2/3_R availability and levels of fasting neuroendocrine hormones (Dunn et al., 2012; Guo et al., 2014). Using the antagonist radiotracer [^18^F]-fallypride, Dunn and colleagues (2012) demonstrated that dopamine D_2/3_R availability in the VS was negatively correlated with IS in a sample of obese and nonobese females. Since radiotracer binding is sensitive to endogenous dopamine at baseline (Laruelle et al., 1997; Verhoeff et al., 2001), one possible explanation for this finding is that persons with reduced IS have less endogenous dopamine occupying D_2/3_R in the VS and therefore more binding of the radiotracer at baseline. It has also been demonstrated with PET that individuals with cocaine addiction have less endogenous dopamine at D_2/3_R in the VS (Martinez et al., 2009). Evidence that individuals with higher insulin resistance also have less endogenous dopamine at D_2/3_R in the VS would support the modulatory role of insulin signaling on dopaminergic brain reward circuits (Daws et al., 2011) and food-seeking behaviors (Pal et al., 2002). However, no in vivo studies have examined how direct estimates of endogenous dopamine levels at D_2/3_R in the VS relate to estimates of IS in humans.

Using PET with particular radioligands for D_2/3_R, it is possible to achieve direct estimates of endogenous dopamine occupying D_2/3_R in humans in vivo. This can be accomplished by comparing the percent change in binding potential (BP_ND_) between a baseline PET scan and a scan under acute dopamine depletion (Laruelle et al., 1997; Verhoeff et al., 2001). Based on the occupancy model, since radiotracer binding to D_2/3_R is sensitive to dopamine levels at baseline, changes in BP_ND_ after dopamine depletion reflect how much dopamine was occupying receptors at baseline (Laruelle et al., 1997; Verhoeff et al., 2001). Acute dopamine depletion can be achieved in humans by inhibiting dopamine synthesis via the tyrosine hydroxylase inhibitor alpha-methyl-para-tyrosine (AMPT). This paradigm has been used to elucidate differences in endogenous dopamine levels occupying D_2/3_R in the striatum of individuals with neuropsychiatric diseases (Martinez et al., 2009).

Our group has developed [^11^C]-(+)-PHNO, the first agonist PET radiotracer for D_2/3_R (Wilson et al., 2005; Graff-Guerrero et al., 2008; Caravaggio et al., 2014). The use of an agonist radiotracer, which should more closely mimic the binding of the endogenous ligand, may offer a more sensitive and functionally significant estimate of endogenous dopamine in humans. Further, we have recently validated the use of [^11^C]-(+)-PHNO to estimate endogenous dopamine levels at D_2/3_R using an AMPT challenge (Caravaggio et al., 2014). Collectively, in vivo human data suggest that this tracer is more sensitive to differences in endogenous dopamine levels than antagonist radiotracers such as [^11^C]-raclopride (Shotbolt et al., 2012; Caravaggio et al., 2014) and thus may be better at elucidating differences in endogenous dopamine levels at D_2/3_R in humans. Using [^11^C]-(+)-PHNO body mass index (BMI) within a nonobese range was found to be positively correlated with BP_ND_ in the VS but not the dorsal striatum (Caravaggio et al., 2015). One potential explanation for this finding is that persons with greater BMI have less endogenous dopamine occupying D_2/3_R in the VS. This previous finding further supports investigating the relationship between IS and endogenous dopamine specifically in the VS as measured with [^11^C]-(+)-PHNO.

Using [^11^C]-(+)-PHNO and an acute dopamine depletion paradigm, we sought to examine for the first time whether estimates of endogenous dopamine at D_2/3_R in the VS of healthy, nonobese humans are related to IS. We hypothesized that persons with reduced IS would have less endogenous dopamine occupying D_2/3_R in the VS at baseline. Healthy participants were evaluated to provide: 1) a proof of concept for the relationship between IS and brain dopamine without the presence of confounding changes that may occur in disease states; and 2) a benchmark for future comparisons in clinical populations. We also sought to determine whether reducing endogenous dopamine with AMPT could lead to changes in IS in healthy individuals. Clarifying the relationship between IS and dopamine levels in the brains of humans in vivo would represent an important first step in understanding the interplay between metabolic health, energy homeostasis, and brain reward circuits in health and disease (Volkow et al., 2013a, 2013b).

## Methods and Materials

### Participants

Data for 9 of the participants, who contribute to the portion of the study estimating endogenous dopamine with PET, were previously reported (Caravaggio et al., 2014). All participants were right-handed and free of any major medical or psychiatric disorder as determined by clinical interview, the Mini International Neuropsychiatric Interview, basic laboratory tests, and electrocardiography. Participants were nonsmokers and were required to have a negative urine screen for drugs of abuse and/or pregnancy at inclusion and before each PET scan. The study was approved by the Research Ethics Board of the Centre for Addiction and Mental Health, Toronto, and all participants provided written informed consent.

### Metyrosine/AMPT Administration

The procedure for AMPT-induced dopamine depletion has been published elsewhere (Verhoeff et al., 2001; Caravaggio et al., 2014). Briefly, dopamine depletion was induced by oral administration of 64mg metyrosine per kilogram of body weight for 25 hours. Independent of weight, no participant was dosed >4500mg. Metyrosine was administered in 6 equal doses at the following times: 9:00 am, 12:30 pm (post 3.5 hours), 5:00 pm (post 8 hours), and 9:00 pm (post 12 hours) on day 1, and 6:00 am (post 21 hours) and 10:00 am (post 25 hours) on day 2. The post AMPT PET scan was scheduled at 12 pm, 28 hours after the initial metyrosine dose. Subjects were under direct observation during AMPT administration and slept overnight in hospital-designated research beds to facilitate the AMPT dosing schedule and monitor for potential side effects. In addition, subjects were instructed to drink at least 4L of fluids during the 2-day admission to prevent formation of AMPT crystals in urine, and fluid intake was monitored to ensure compliance. In addition, to alkalinize the urine, which increases AMPT solubility, sodium bicarbonate (1.25g) was given orally at 10:00 pm on the evening before day 1 and at 7:00 am on day 1 of administration.

### Fasting Plasma Data

Participants were requested to refrain from eating and drinking fluids except water for 10 to 12 hours prior to the collection of blood work, collected at 9:00 am. For the participants who provided PET scans (n=11), fasting blood work was collected on the day of the baseline PET scan. Twenty-five healthy participants (9 females, mean age=31±11, BMI: 22–28) provided fasting blood work (9:00 am) at baseline and after receiving 5 doses of AMPT. For 13 of these subjects, it was possible to collect the blood work 24 hours apart. For the remainder of subjects, 4 provided blood work 6 to 7 days apart, 4 provided 10 to 14 days apart, and 2 provided 36 to 43 days apart. Blood for glucose measurement was collected in a 4-mL grey stoppered tube containing sodium fluoride as a preservative and potassium oxalate as an anticoagulant. Plasma was assayed for glucose on the EXL 200 Analyzer (Siemens) using an adaptation of the hexokinase-glucose-6-phosphate dehydrogenase method. Blood for insulin measurement was collected in a 6-mL red stoppered tube with no additives. Serum was analyzed on an Access 2 Analyzer (Beckman Coulter) using a paramagnetic particle, chemiluminescent immunoassay for the quantitative determination of insulin levels in human serum. The IS index for glucose disposal was estimated for each subject from fasting plasma glucose and insulin using the Homeostasis Model Assessment II (HOMA2), calculated with the University of Oxford HOMA2 calculator (v2.2.2; http://www.dtu.ox.ac.uk/homacalculator/) (Wallace et al., 2004). Estimates of IS achieved using the HOMA2 are highly correlated with those achieved with the hyperinsulinemic-euglycemic clamp method (Matthews et al., 1985; Levy et al., 1998).

### PET Imaging

Participants underwent 2 [^11^C]-(+)-PHNO PET scans, one under baseline conditions and another at 25 hours following AMPT-induced dopamine depletion. The radiosynthesis of [^11^C]-(+)-PHNO and the acquisition of PET images have been described in detail elsewhere (Wilson et al., 2000, 2005; Graff-Guerrero et al., 2010). Briefly, images were acquired using a high resolution, head-dedicated PET camera system (CPS-HRRT; Siemens Molecular Imaging) measuring radioactivity in 207 brain slices with a thickness of 1.2mm each. The in-plane resolution was ~2.8mm full-width at half-maximum. Transmission scans were acquired using a ^137^Cs (T_1/2_ = 30.2 yr, E = 662 KeV) single-photon point source to provide attenuation correction, and the emission data were acquired in list mode. The raw data were reconstructed by filtered-back projection. For baseline [^11^C]-(+)-PHNO scans (n=11), the mean radioactivity dose was 9 (±1.5) mCi, with a specific activity of 1087 (±341) mCi/µmol and an injected mass of 2.2 (±0.4) µg. For the dopamine-depleted scans (n=9), the mean radioactivity dose was 9 (±1.6) mCi, with a specific activity of 1044 (±310) mCi/µmol and an injected mass of 2.1 (±0.4) µg. There was no difference in mean radioactivity dose (*t*(8)=0.98, *P*=.36), specific activity (*t*(8)=1.09, *P*=.31), or mass injected (*t*(8)=-0.61, *P*=.56) between the baseline and dopamine depletion scans (n=9). [^11^C]-(+)-PHNO scanning data were acquired for 90 minutes postinjection. Once scanning was complete, the data were redefined into 30 frames (1–15 of 1-minute duration and 16–30 of 5-minute duration).

### Image Analysis

The region of interest (ROI)-based analysis for [^11^C]-(+)-PHNO has been described in detail elsewhere (Graff-Guerrero et al., 2008; Tziortzi et al., 2011). Briefly, time activity curves (TACs) from ROIs were obtained from the dynamic PET images in native space with reference to each subject’s co-registered MRI image. The co-registration of each subject’s MRI to PET space was attained using the normalized mutual information algorithm (Studholme et al., 1997), as implemented in SPM2 (SPM2, Wellcome Department of Cognitive Neurology, London; http://www.fil.ion.ucl.ac.uk/spm). The TACs were analyzed using the Simplified Reference Tissue Method (Lammertsma and Hume, 1996) using the cerebellum as the reference region, to derive a quantitative estimate of binding: binding potential relative to nondisplaceable compartment (BP_ND_), as defined by consensus nomenclature for in vivo imaging of reversibly binding radioligands (Innis et al., 2007). The basis function implementation of the Simplified Reference Tissue Method (Gunn et al., 1997) was applied to the dynamic PET images to generate parametric voxel-wise BP_ND_ maps using PMOD (v2.7, PMOD Technologies, Zurich, Switzerland). The range wherein the basis functions were generated (K_2_a min - K_2_a max) was 0.006 to 0.6. These images were spatially normalized into MNI brain space by Nearest Neighbour interpolation with a voxel size fixed in 2×2 × 2mm^3^ using SPM2. Regional BP_ND_ estimates were then derived from ROIs defined in MNI space. The VS and dorsal striatum (dorsal caudate, hereafter caudate and dorsal putamen, hereafter putamen) were defined according with Mawlawi et al. (2001).

### Estimating Endogenous Dopamine Levels

Estimates of endogenous dopamine levels at D_2/3_R were based on an occupancy model in which binding of radiotracers like [^11^C]-(+)-PHNO for D_2/3_R is sensitive to dopamine levels (Laruelle et al., 1997; Verhoeff et al., 2001; Cumming et al., 2002). It is assumed with this model that: 1) baseline D_2/3_R BP_ND_ is confounded by endogenous dopamine, that is, the higher the concentration of dopamine, the lower the value of D_2/3_R BP_ND_; 2) D_2/3_R BP_ND_ under depletion more accurately reflects the true number status of D_2/3_R; and 3) the fractional increase in D_2/3_R BP_ND_ after dopamine depletion [ie, 100*(Depletion BP_ND_ – Baseline BP_ND_)/ Baseline BP_ND =_ %ΔBP_ND_] is linearly proportional to baseline dopamine concentration at D_2/3_R, provided the process of dopamine depletion does not change the number and affinity of D_2/3_R. Thus, the %ΔBP_ND_, under appropriate assumptions, is considered a semiquantitative index of endogenous dopamine levels at D_2/3_R (Verhoeff et al., 2001). Based on our previous analyses, we were unable to estimate endogenous dopamine in the substantia nigra, nor were we able to reliably estimate endogenous dopamine in the hypothalamus and ventral pallidum for all subjects (Caravaggio et al., 2014). Therefore, these ROIs were not investigated in the current analysis.

### Statistical Analysis

Our a priori hypothesis was to examine the relationship between IS and endogenous dopamine in the VS. We conducted exploratory analyses between IS and endogenous dopamine in the rest of the striatum: caudate, putamen, and globus pallidus.

Relationships between baseline BP_ND_ and IS were explored in an ROI only to clarify any findings with endogenous dopamine levels (if any). Statistical analyses were conducted using SPSS (v.12.0; SPSS, Chicago, IL) and GraphPad (v.5.0; GraphPad Software, La Jolla, CA). Normality of variables was determined using the D’Agostino-Pearson test. The significance level for all testes was set at *P*<.05 (2-tailed).

## Results

Eleven healthy, nonobese and nondiabetic individuals (3 female) participated in the PET portion of the study; a subset of these data have been previously reported upon (Table 1) (Caravaggio et al., 2014). Within the full sample of subjects (n=11), examination of correlations between participant metabolic variables revealed that age was positively correlated with waist circumference (*r*(9)=.76, *P*=.007), and waist circumference was positively correlated with fasting levels of insulin (*r*(9)=.80, *P*=.003) (Table 2).

**Table 1.** Participant Demographics

	Baseline PET Participants (n=11)	AMPT-PET Participants (n=9)
Age (years)	29 (8)	29 (9)
range:	20–43	20–43
Fasting glucose (mmol/L)	5 (0.3)	5 (0.3)
range:	4.3–5.3	4.3–5.3
Fasting insulin (pmol/L)	31 (25)	34 (26)
range:	15–101	15–101
Insulin sensitivity (%S)	211 (70)	197 (70)
range:	53–276	53–276
Body Mass Index (kg/m2)	25 (2.4)	25 (2.4)
range:	22–28	22–28
Waist circumference (cm)	35 (6)	36 (7)
range:	27–52	27–52

Values indicate means with standard deviation in parentheses. Abbreviations: AMPT, alpha-methyl-para-tyrosine; PET, positron emission tomography.

**Table 2.** Pearson Correlations between Metabolic Variables

	Age	BMI	Waist Circumference	Fasting Glucose	Fasting Insulin
Insulin sensitivity	-0.179 (*P*=.599)	-0.571 (*P* =.067)	-0.602^**†**^ (*P* =.050)	-0.517 (*P* =.103)	-0.926*** (*P* =.0001)
Fasting insulin	0.422 (*P* =.196)	0.529 (*P* =.095)	0.795** (*P* =.003)	0.598^**†**^ (*P* =.052)	
Fasting glucose	0.420 (*P* =.199)	0.063 (*P* =.855)	0.516 (*P* =.104)		
Waist circumference	0.756** (*P* =.007)	0.466 (*P* =.149)			
Body Mass Index	0.050 (*P* =.883)				

^**†**^Correlation is at trend level of significance: 0.05 (2-tailed).

**Correlation is significant at the 0.01 level (2-tailed).

***Correlation is significant at the 0.001 level (2-tailed).

**Figure 1.**
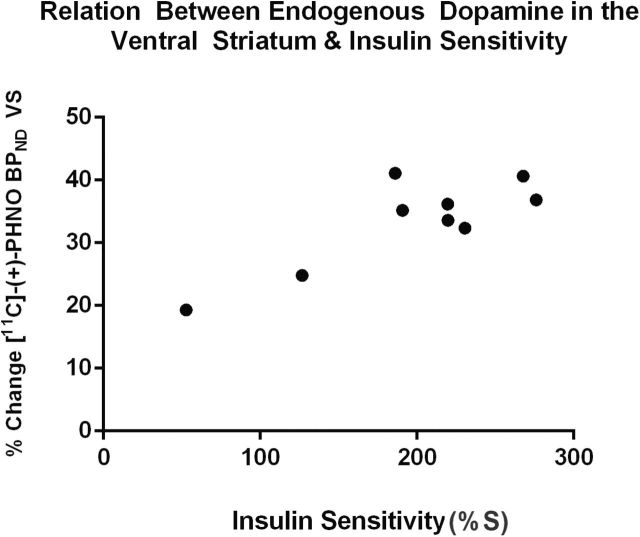
Relationship between estimated insulin sensitivity (IS) and endogenous dopamine at D_2/3_ receptors (D_2/3_R) in the ventral striatum (VS) of 9 healthy persons.

Nine of the 11 subjects provided both a baseline PET scan as well as a scan under acute AMPT-induced dopamine depletion; this provided estimates of endogenous dopamine occupying D_2/3_R in the VS at baseline (ie, the percent change in [^11^C]-(+)-PHNO BP_ND_ before and after dopamine depletion). Estimated baseline dopamine occupancy of D_2/3_R in the VS was positively correlated with IS (*r*(7)=.84, *P*=.005) (Figure 1), a correlation that remained after statistically controlling independently for age (*r*(6)=.86, *P*=.007), BMI (*r*(6)=.72, *P*=.04), waist circumference (r(6)=.75, *P*=.03), and plasma levels of AMPT (*r*(6)=.84, *P*=.009). Concurrently, estimated baseline dopamine occupancy of D_2/3_R in the VS was negatively correlated with fasting insulin levels (*r*(7)=-.85, *P*=.004) but was not correlated with fasting levels of glucose (*r*(7)=-.49, *P*=.18). Dopamine occupancy in the VS was not correlated with BMI (*r*(7)=.09, *P*=.80) or waist circumference (*r*(7)=-.30, *P*=.41).

Notably, the above correlations with estimated baseline dopamine occupancy of D_2/3_R were driven primarily by dopamine occupancy in the right VS but not the left VS. Specifically, dopamine occupancy in the left VS was not correlated with IS (*r*(7)=.41, *P*=.28), fasting levels of insulin (*r*(7)=-.46, *P*=.22), or glucose (*r*(7)=-.33, *P*=.39), whereas dopamine occupancy in the right VS was positively correlated with IS (*r*(7)=.75, *P*=.01), negatively correlated with fasting levels of insulin (*r*(7)=-.73, *P*=.02), and not correlated with levels of glucose (*r*(7)=-39., *P*=.31).

**Figure 2.**
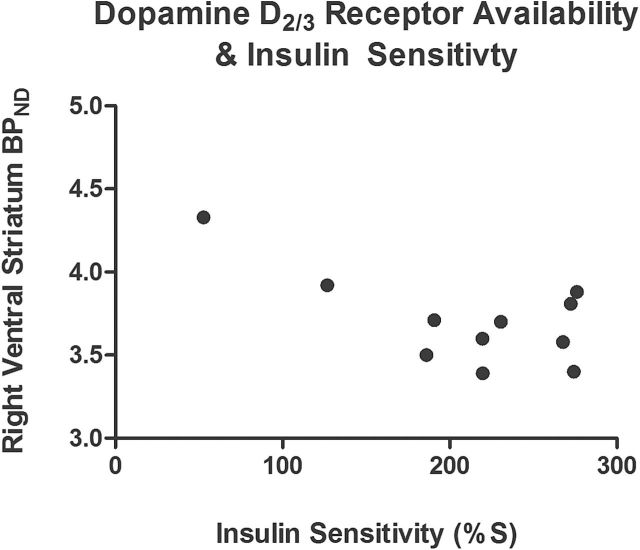
Relationship between baseline dopamine D_2/3_ receptor (D_2/3_R) availability – [^11^C]-(+)-PHNO BP_ND_ – and estimated insulin sensitivity (IS) in 11 healthy persons.

Within the full sample of subjects (n=11), baseline [^11^C]-(+)-PHNO BP_ND_ in the right VS was negatively correlated with estimated IS (*r*(9)=-.65, *P*=.02) (Figure 2). Thus, participants with the lowest levels of endogenous dopamine occupying D_2/3_R had the highest BP_ND_ at baseline, consistent with reduced competition for tracer binding by endogenous dopamine with decreased IS. Concurrently, fasting levels of insulin were positively correlated with [^11^C]-(+)-PHNO BP_ND_ in the right VS (*r*(9)=.77, *P*=.006), while there was no correlation with fasting glucose levels (*r*(9)=.27, *P*=.43). Notably, [^11^C]-(+)-PHNO BP_ND_ in the left VS was not correlated with IS (*r*(9)=-.35, *P*=.29) or fasting levels of insulin (*r*(9)=.53, *P*=.09) and glucose (*r*(9)=.08, *P*=.81).

Exploratory analyses revealed that estimated IS was not correlated with estimates of endogenous dopamine at D_2/3_R in the caudate (*r*(7)=.47, *P*=.20), putamen (*r*(7)=.52, *P*=.15), or globus pallidus (*r*(7)=.33, *P*=.40). There were also no correlations between estimates of dopamine occupancy in these regions and fasting levels of insulin or glucose, as well as BMI and waist circumference (all *P*>.05; data not shown).

**Figure 3.**
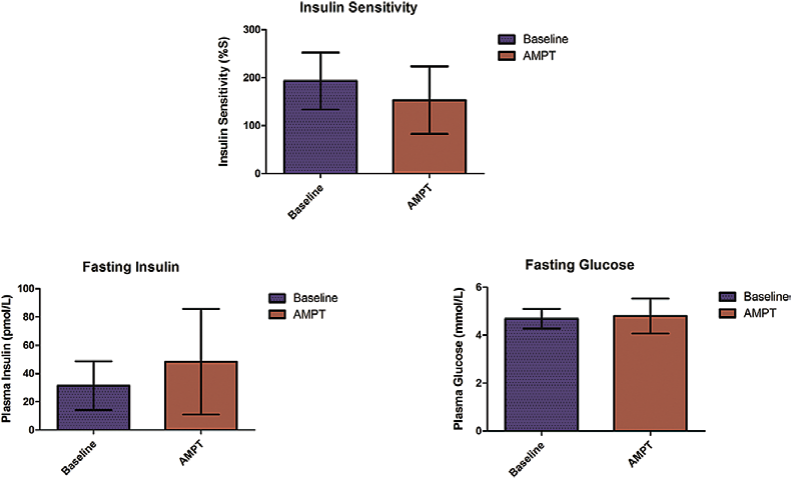
Effect of acute dopamine depletion via alpha-methyl-para-tyrosine (AMPT) on estimated insulin sensitivity (IS), and fasting plasma levels of insulin and glucose, in 25 healthy persons (error bars represent SD). For 8 subjects, their post depletion IS values went against the general trend: 6 increased and 2 remained the same.

To examine how reducing endogenous dopamine affects IS, 25 healthy controls (mean age=31±11; 9 female) also provided fasting plasma levels of insulin and glucose before and after AMPT dopamine depletion. AMPT significantly increased plasma levels of fasting insulin (*t*(24)=-2.62, *P*=.01) while not significantly altering plasma levels of fasting glucose (*t*(24)=-0.93, *P*=.36). Of note, AMPT significantly decreased estimated IS (*t*(24)=2.82, *P*=.01) (Figure 3). Removing those subjects who had more than a 2-week interval between collection of blood work did not significantly change the aforementioned results (data not shown).

## Discussion

Using the agonist radiotracer [^11^C]-(+)-PHNO and an acute dopamine depletion paradigm, we demonstrate for the first time that IS is positively correlated with endogenous dopamine levels at D_2/3_R in the VS. In the absence of obesity or overt glucose dysregulation, lower endogenous dopamine levels in the VS are associated with reduced IS. This novel finding is in line with previous in vivo PET studies examining baseline D_2/3_R availability in the VS of obese persons (Dunn et al., 2012) and supports previous postmortem human findings (Lackovic et al., 1990) as well as preclinical findings in animals (Murzi et al., 1996; O’Dell et al., 2014). In line with the PET findings, experimentally reducing endogenous dopamine in a sample of healthy persons was associated with reduced IS.

Evidence suggests that brain insulin resistance co-occurs with peripheral insulin resistance, with insulin-resistant individuals demonstrating reduced glucose metabolism in the VS and prefrontal cortex in response to peripheral insulin (Anthony et al., 2006). Interestingly, central D_2/3_R agonism in rodents can increase glucose concentrations in the periphery, not just in the brain (Arneric et al., 1984; Saller and Kreamer, 1991). Within this context, it warrants comment that bromocriptine, a nonspecific dopamine receptor agonist, is indicated for the treatment of diabetes (Grunberger, 2013; Kumar et al., 2013). Thus, centrally altering dopamine/insulin receptor functioning in the VS of humans may have clinical implications in the treatment of metabolic disorders. It should be noted that while dopamine in the accumbens is altered by changes in blood glucose in response to hyperinsulinemia, this relationship may be complex, with timing (acute vs chronic) and dose (physiological vs supraphysiological) effects both appearing to be important (Bello and Hajnal, 2006).

Limitations of our current study include not sampling individuals with glucose dysregulation; accordingly, clinical implications specific to overt cardiometabolic pathology are difficult to comment on. It is suggested that future studies examine how different degrees of glucose dysmetabolism (eg, insulin resistance, prediabetes, diabetes) are related to endogenous dopamine levels and dopamine release in the VS of humans. In addition, future studies should examine whether these values alter in the face of treatment for metabolic deficits. Moreover, it is important to examine across a spectrum of glucose dysregulation in humans how dopamine concentrations and functioning in the VS relate to mood, motivation, and reward processing. Finally, our sample in the current study is small. While we did not explicitly control for multiple comparisons, it is important to note that the observed relationship between IS and estimated endogenous dopamine in the VS would survive Bonferroni correction (corrected *P* value threshold for significance: *P*=.01 (0.05/4 ROIs). Future AMPT studies examining the relationship between endogenous dopamine in the brain and IS should attempt to employ larger sample sizes. Because of our small sample size, we refrained from exploring relationships between baseline [^11^C]-(+)-PHNO BP_ND_ and IS in ROIs other than the VS. In particular, future [^11^C]-(+)-PHNO studies using larger sample sizes should examine the relationship between IS and baseline BP_ND_ in the substantia nigra and hypothalamus: regions where 100% of the [^11^C]-(+)-PHNO BP_ND_ signal is due to D_3_R vs D_2_R (Searle et al., 2010; Tziortzi et al., 2011). To our knowledge, studies have not examined if there is a differential relationship between central D_3_R vs D_2_R expression with peripheral insulin resistance in animals or humans. This warrants investigation, since D_3_R may play a role in insulin secretion in the periphery (Ustione and Piston, 2012), and D_3_R knockout mice have been characterized as having an obesity-prone phenotype (McQuade et al., 2004).

What is the relationship between insulin, changes in dopamine concentrations, and food reward? Changes in insulin appear to modify functioning of the mesolimbic dopamine system, affecting feeding and food reward (Figlewicz et al., 2006; Labouebe et al., 2013). It has been suggested that insulin can inhibit dopamine neurons in the ventral tegmental area (VTA) and thus reduce dopamine release into the accumbens (Palmiter, 2007). Notably, acute insulin injections into the VTA have been shown to inhibit overeating of sweetened high-fat foods in sated rodents without altering hungry feeding (Mebel et al., 2012). Moreover, hypoinsulinemic rodents demonstrate increased feeding related to altered functioning of the nucleus accumbens (Pal et al., 2002). Data in healthy rodents suggest that peripheral insulin injections can increase dopamine release in the nucleus accumbens (Potter et al., 1999), and insulin per se may be rewarding (Jouhaneau and Le Magnen, 1980; Castonguay and Dubuc, 1989). Thus, the exact mechanisms by which acute or chronic insulin receptor activation affects the mesolimbic dopamine system and dopamine levels therein are not entirely clear. In addition, it is unclear how these systems may change in healthy metabolic states vs those that are diseased.

Several studies have examined how insulin affects DAT and reward-related behaviors to drugs of abuse that act on DAT, such as cocaine and amphetamine (Daws et al., 2011). For example, hypoinsulinemic rodents self-administer less amphetamine (Galici et al., 2003), while increasing insulin in the accumbens enhances cocaine-induced impulsivity (Schoffelmeer et al., 2011). However, while the molecular pathways by which insulin can alter DAT function and expression are known, mixed results have been observed across studies employing either acute or chronic insulin manipulations for the striatum (Galici et al., 2003; Owens et al., 2005; Sevak et al., 2007; Williams et al., 2007; Schoffelmeer et al., 2011; Owens et al., 2012; O’Dell et al., 2014) and VTA (Figlewicz et al., 1996, 2003; Mebel et al., 2012). Many of these studies have not differentially examined how insulin affects DAT in the dorsal striatum vs the VS, or the accumbens core vs shell. This may be a potential source of discrepancy, since the expression, regulation, and function of the DAT may be different in different striatal subregions (Nirenberg et al., 1997; Siciliano et al., 2014). To our knowledge, no in vivo human brain imaging study has investigated the relationship between insulin resistance and striatal DAT availability. Findings regarding the relationship between BMI and striatal DAT availability in humans have been mixed (Chen et al., 2008; Thomsen et al., 2013; van de Giessen et al., 2013), although these studies have not examined the VS. Interestingly, amphetamine users report a high incidence of childhood obesity and eating psychopathology (Ricca et al., 2009), further highlighting the important behavioral and neurochemical overlaps between food and drug reward (Volkow et al., 2013b).

The current finding that lower IS is associated with reduced dopamine in the VS could have implications for theories of food and drug addiction. It has been suggested that increased BMI and overeating behavior are related to reduced presynaptic dopamine synthesis capacity in the striatum of healthy humans (Wilcox et al., 2010; Wallace et al., 2014). Data from Wang and colleagues (2014) suggest that obese individuals demonstrate attenuated dopamine release in the VS in response to calorie consumption compared with nonobese persons. Moreover, using SPECT, it has been suggested that obese females demonstrate reduced striatal dopamine release in response to amphetamine (van de Giessen et al., 2014). This may well mirror the blunted VS dopamine release seen in diabetic rodents and in persons with drug addiction in response to psychostimulants (Volkow et al., 2009). It will be important to elucidate whether persons with diabetes also display a blunting of striatal dopamine release in response to food, food cues, and/or psychostimulants. Collectively, in vivo brain imaging studies in humans suggest that obesity and perhaps insulin resistance are associated with reduced dopamine synthesis, release, and endogenous tone in the VS.

While we did not find any relationship between IS and levels of endogenous dopamine in the dorsal striatum, it is important to highlight that several animal studies have reported alterations in dorsal striatal dopamine and functioning of neurons in the substantia nigra in relation to insulin resistance (Morris et al., 2011). Notably, in humans dopamine release in response to food in the dorsal striatum has been found to be correlated with ratings of meal pleasantness (Small et al., 2003). Perhaps, reduced IS affects VS dopamine functioning first, with changes in dorsal striatal dopamine functioning only evident with greater insulin resistance. It is possible that the present study was underpowered and/or did not sample a wide enough range of IS to detect an effect in the dorsal striatum.

These data have important implications for those neuropsychiatric disorders in which insulin resistance may be co-morbid or concurrent. For example, several lines of evidence suggest links between insulin resistance and the development of Parkinson’s disease (Santiago and Potashkin), Alzheimer’s disease (Willette et al., 2014), and depression (Pan et al., 2010). Consistent with the hypothesis that insulin resistance may be associated with decreased striatal dopamine, it is tempting to speculate that lower IS could confer protective effects on psychosis in persons with schizophrenia. For example, in Chinese first-episode, never-medicated persons with schizophrenia, greater insulin resistance was correlated with reduced severity of positive symptoms (Chen et al., 2013). It is well established that persons with schizophrenia, as well as their unaffected relatives (Fernandez-Egea et al., 2008), are more likely to have metabolic abnormalities; this has been found prior to antipsychotic use and after controlling for lifestyle habits (Kirkpatrick et al., 2012). Moreover, differences in glucose tolerance may differentiate subgroups of persons with schizophrenia characterized by different courses of symptom severity (Kirkpatrick et al., 2009). In the context of these findings, combined with the historical observation that insulin-induced comas can ameliorate psychotic symptoms (West et al., 1955), it is appealing to speculate that central insulin signaling on dopamine neurons may play a role in the pathology and treatment of schizophrenia (Lovestone et al., 2007). Future PET studies exploring the interaction between psychopathology and insulin resistance on central dopamine levels certainly appear warranted.

In conclusion, using PET and an acute dopamine depletion challenge, we have demonstrated for the first time that estimates of IS are related to levels of endogenous dopamine at D_2/3_R in the VS of healthy humans. Furthermore, acutely reducing endogenous dopamine in healthy persons can alter estimated IS. Taken together, these findings represent an important preliminary step in elucidating how metabolic status may interface with major mental illnesses such as schizophrenia.

## Statement of Interest

Dr. Nakajima reports having received grants from Japan Society for the Promotion of Science and Inokashira Hospital Research Fund and speaker’s honoraria from GlaxoSmith Kline, Janssen Pharmaceutical, Pfizer, and Yoshitomiyakuhin within the past 3 years. Dr. Graff-Guerrerro currently receives research support from the following external funding agencies: Canadian Institutes of Health Research, the U.S. National Institute of Health, and the Mexico Instituto de Ciencia y Tecnologıa para la Capital del Conocimiento en el Distrito Federal (ICyTDF). He has also received professional services compensation from Abbott Laboratories, Gedeon-Richter Plc, and Lundbeck; grant support from Janssen; and speaker compensation from Eli Lilly. Dr. Remington has received research support, consulting fees, or speaker’s fees from the Canadian Diabetes Association, the Canadian Institutes of Health Research, Hoffman-La Roche, Laboratorios Farmacéuticos Rovi, Medicure, Neurocrine Biosciences, Novartis Canada, Research Hospital Fund–Canada Foundation for Innovation, and the Schizophrenia Society of Ontario. The other authors have no competing interests to disclose.
